# Anatomical variations of the calcaneofibular ligament in human foetuses

**DOI:** 10.1038/s41598-023-37799-2

**Published:** 2023-07-07

**Authors:** K. Ruzik, B. Gonera, M. Podgórski, N. Zielinska, A. Balcerzak, Ł. Olewnik

**Affiliations:** 1 Department of Anatomical Dissection and Donation, Medical University of Lodz, Lodz, Poland; 2 Department of Interventional Radiology, Medical University of Lodz, Lodz, Poland

**Keywords:** Anatomy, Musculoskeletal system

## Abstract

Ligaments anatomy often show a huge anatomy variations between species and individuals. For example calcaneofibular ligaments (CFL) characterize the great variability of morphological shape or presence of additional bands. The aim of this study was to propose first anatomical classification of CFL concerning on human fetuses. We investigated thirty spontaneously-aborted human fetuses aged 18–38 weeks of gestation at death. Sixty lower limbs (30 left and 30 right) fixed in 10% formalin solution were examined. The morphological variability of CFL was assessed. Four types of CFL morphology were observed. Type I was characterized by a band shape. This was the most common type, occurring in 53% of all cases. Based on our study we are proposing a classification based on four morphological types of CFL. Types 2 and 4 are further divided into subtypes. Present classification may be useful to better understand the anatomical development of ankle joint.

## Introduction

The lateral collateral ligament complex (LCL) of the ankle joint consists of the calcaneofibular (CFL), anterior talofibular (ATFL) and the posterior talofibular ligaments (PTFL). Recent anatomical studies have described the relationship between the inferior band of ATFL and CFL and proposed the existence of a lateral fibulotalocalcaneal ligament (LFTCL) complex, which plays an essential role in maintaining lateral ankle stability^1,2^. According to classical anatomy textbooks, the CFL origin is located on the tip of the lateral malleolus and courses posteroinferiorly and medially to insert onto the lateral surface of the calcaneus.

Although many studies describe the anatomical types of CFL, these have all focused on adult human cadavers. Their classifications were based on the shape of the main band and the presence of additional fibers.

One of the most common musculoskeletal injuries, particularly during sporting activities, is ankle sprain. The most common mechanism of injury is inversion of the foot. Although the most common ruptured ligament is the ATFL, 20% of sprains involve the CFL rupture combined with ATFL.

The aim of this study was to determine the morphological variability of the CFL and thus establish the first classification of CFL types in human fetuses.The results can be compared with the spectrum of morphological variability in adults.

## Materials and methods

Thirty spontaneously-aborted human fetuses (11 male, 19 female) aged 18–38 weeks of gestation at death were examined. The fetuses were donated to the Chair of Anatomy and Histology before 1998.The study was performed in accordance with the legal procedures in force in Poland and in accordance with the Donation Corpse program for both adults and fetuses. The age of the fetuses was determined from the head and craniosacral measurements. Permission for the study was given by the Local Bioethics Commission (agreement no. RNN/242/22/KE).

### Protocol of anatomical dissection

Sixty paired lower limbs of human fetuses were dissected by the same orthopedic surgeon. The protocol for the dissection of the lateral compartment of the leg and lateral site of the foot was described previously^3,4^. Dissection started with incision of the skin placed 2 cm above the lateral mallelous; the skin, subcutaneous and superficial fascia were removed towards the lateral ankle. After the fibularis longus muscle and fibularis brevis tendons were identified, the skin and subcutaneous tissue of the lateral compartment of the foot were removed. Following this, peroneal tendons were cut below the lateral mallelous and lifted to visualize CFL. The following characteristics were recorded:

1. Morphological type of CFL.
2. Length of CFL and additional bands if present.
3. The thickness and width of the CFL, and any additional bands that were present,at three points; origin, in the middle and insertion.

The foot was positioned in a neutral position for all measures. Measurements were made with an electronic digital caliper (Mitutoyo Corporation, Kawasaki-shi, Kanagawa, Japan). Each measurement was performed twice by independent researchers with an accuracy of up to 0.01 mm.

### Statistical analysis

Statistical analysis was performed using Statistica 13 software [TIBCO Software Inc. (2017). Statistica, http://statistica.io]. The Shapiro–Wilk test indicated that continuous variables had a non-normal distribution; therefore nonparametric tests were used. CFL types were compared using the Mann–Whitney test (for two groups) and the Kruskal–Wallis test by ranks with a dedicated *posthoc* test (more than two groups). A p-value of less than 0.05 was considered significant; this value was modified for multiple testing with Bonferroni correction. Results are presented as mean and standard deviation unless otherwise stated.

## Results

**Figure 1:**
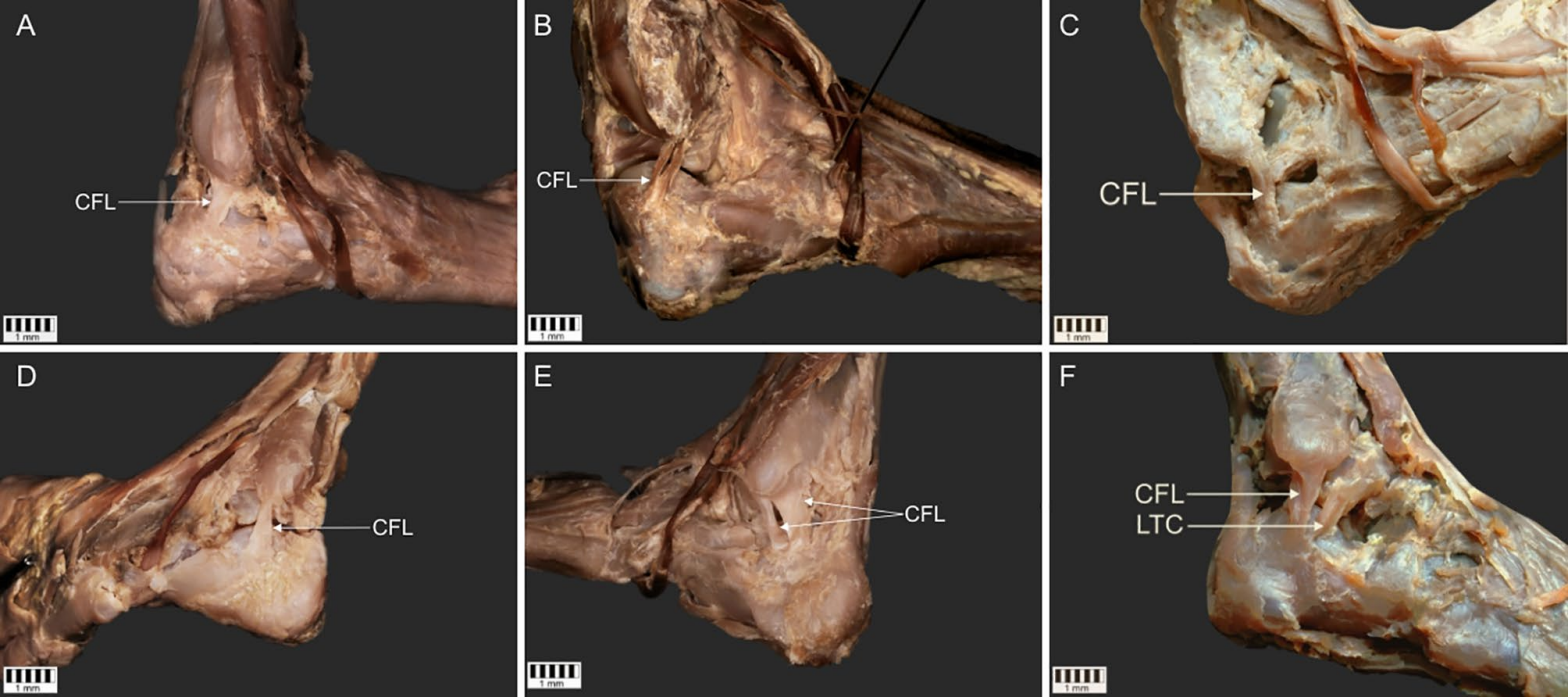
Types of the Calcaneofibular ligament morphology. *CFL* calcaneofibular ligament, *LTC* lateral talocalcaneal ligament.

**Figure 2:**
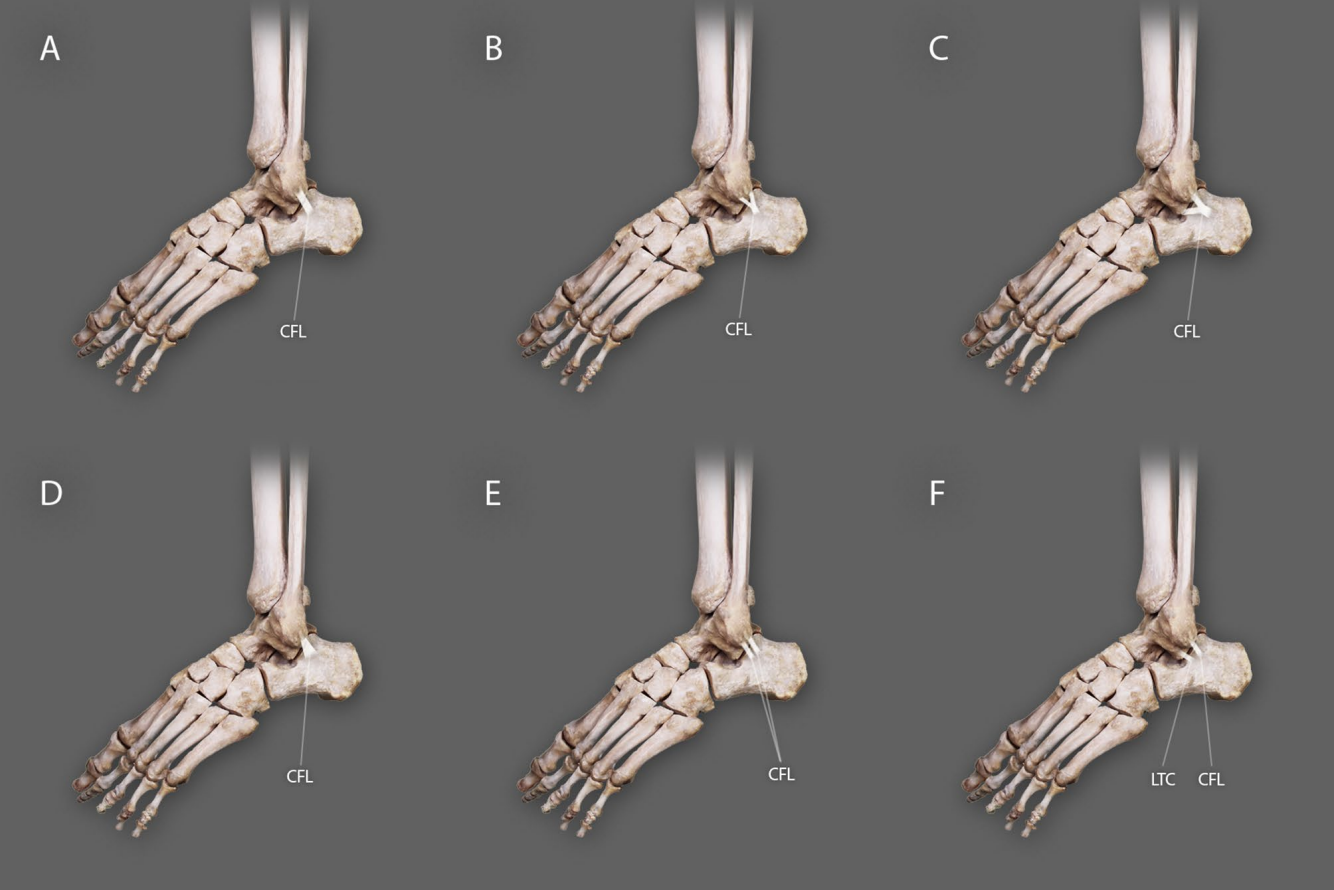
Schematic drawing of CFL morphological types. *CFL* calcaneofibular ligament, *LTC* lateral talocalcaneal ligament.

The CFL was present in 100% of 60 dissected lower limbs. Its prevalence is presented in Table 1. The following four types were distinguished with subtypes (Figs. 1, 2).

- Type I-band-shaped; the origin was located on the lateral malleolus and the insertion on the lateral surface of the calcaneus bone. It was present in 32 cases (53%) (Fig. 1a).
- Type II-Y-shaped; present in 11 cases (18%). In three cases (5%), both arms of the ligament were attached to the lateral malleolus (subtype A) and in the remaining cases (13%), one was attached to the lateral malleolus and the other one to the talus (subtype B). In both subtypes, the insertion was located on the lateral surface of the calcaneus bone (Fig. 1b,c).
- Type III–V-shaped; the origin was located on the lateral malleolus with the insertion on the lateral surface of the calcaneus bone. The width of the insertion point was at least 1.5 mm greater than the width of the origin. It was present in 10 cases (17%) (Fig. 1d).
- Type IV-double band; main band origin was located on the lateral malleolus and an insertion on lateral surface of calcaneus bone. It was observed in seven cases (12%). Two subtypes were recorded: subtype A with additional CFL originating on the lateral malleolus (two cases, 4%), and subtype B with presence of lateral talocalcaneal ligament originating on the talus bone (five cases; 8%). Both subtypes inserted on the lateral surface of the calcaneus bone (Fig. 1e,f).

**Table 1:** Comparison of morphological data types of the CFL with regard to sex and side of body.

	Female		Male		Total
	Female	Male	Right side	Left side	
Type I	8 (13.4%)	12 (20%)	6 (10%)	6 (10%)	32 (53%)
Type II	6 (10%)	2 (3.3%)	1 (1.7%)	2 (3.3%)	11 (18%)
Type III	3 (5%)	4 (6.7%)	2 (3.3%)	1 (1.7%)	10 (17%)
Type IV	2 (3.3%)	1 (1.7%)	2 (3.3%)	2 (3.3%)	7 (12%)
Total	19	19	11	11	60

The mean cranio-sacral (CS) length in the whole group was 174.1 (± 31.1). No difference in CS length was observed between types (type I: 167.5 ± 26.9; type II: 196.7 ± 38.9; type III: 170.1 ± 27.4; type IV: 174.4 ± 31.2; p = 0.1430).

The differences between CFL types regarding the origin and distal attachment are given in Table 2. The statistical analysis only tested the variables that were observed in the majority of cases. Table 3 presents differences in morphological parameters between sexes and body sides. According to post hoc analysis:

- Thickness of main band at distal attachment point was bigger in type III than in other types.
- Width of main band in the center was bigger in types II and III than in type IV.
- Width of main band at origin in type III than in type IV.

**Table 2:** The differences between CFL types regarding the origin and distal attachment.

Parameter	Type				P
	I	II	III	IV	
Width of main band at origin	1.40 (0.58)	1.69 (0.55)	2.17 (0.57)	1.02 (0.53)	0.0023
Thickness of main band at origin	0.22 (0.12)	0.36 (0.3)	0.31 (0.11)	0.20 (0.12)	0.0433
Width of second band at origin		1.23 (0.63)	1.57 (0)	0.96 (0.6)	0.4366
Thickness of second band at origin		0.20 (0.08)	0.31 (0)	0.15 (0.11)	0.2986
Width of main band in the center	1.12 (0.47)	1.51 (0.48)	1.65 (0.58)	0.90 (0.3)	0.0043
Thickness of main band in the center	0.17 (0.08)	0.26 (0.08)	0.26 (0.09)	0.16 (0.12)	0.0079
Width of second band band in the center				0.66 (0.42)	
Thickness of second band band in the center				0.10 (0.08)	
Width of main band at distal attachment point	1.25 (0.49)	1.40 (0.40)	2.84 (1.05)	1.12 (0.47)	0.0016
Thickness of main band at distal attachment point	0.26 (0.15)	0.38 (0.25)	0.30 (0.06)	0.20 (0.15)	0.1229
Width of second band at distal attachment point				0.97 (0.72)	
Thickness of second band at distal attachment point				0.12 (0,09)	
Length of main band or length to split in Y type	3.39 (0.99)	2.39 (0.8)	3.78 (0.79	2.70 (1.29)	0.0069
Lenght of second band				3.43 (1.11)	
Lenght of posterior arm to split point of the ligament in Y type		1.51 (0.45)			
Lenght of anterior arm to split point of the ligament in Y type		2.44 (0.94)			

Significant p according to Bonferroni correction is 0.005.

**Table 3:** Differences in morphometric parameters between sexes and body sides.

Parameter	Body side		P value	Sex		P value
	R	L		F	M	
Width of main band at origin	1.49 (0.61)	1.56 (0.69)	0.7159	1.65 (0.65)	1.29 (0.58)	0.0496
Thickness of main band at origin	0.26 (0.22)	0.25 (0.12)	0.3244	0.27 (0.19)	0.23 (0.14)	0.5422
Width of second band at origin	1.08 (0.66)	1.24 (0.55)	0.4828	1.26 (0.63)	0.99 (0.58)	0.3423
Thickness of second band at origin	0.17 (0.10)	0.21 (0.09)	0.4328	0.17 (0.09)	0.21 (0.09)	0.3637
Width of main band in the center	1.19 (0.41)	1.32 (0.62)	0.4508	1.33 (0.53)	1.13 (0.51)	0.1582
Thickness of main band in the center	0.29 (0.21)	0.28 (0.12)	0.6256	0.31 (0.18)	0.24 (0.14)	0.1347
Width of second band band in the center	0.53 (0.47)	0.84 (0.33)	0.3768	0.70 (0.45)	0.64 (0.46)	0.5959
Thickness of second band band in the center	0.06 (0.05)	0.16 (0.08)	0.2159	0.07 (0.05)	0.12 (0.09)	0.5959
Width of main band at distal attachment point	1.43 (0.74)	1.63 (0.93)	0.4204	1.60 (0.86)	1.40 (0.81)	0.2727
Thickness of main band at distal attachment point	0.29 (0.21)	0.28 (0.12)	0.6256	0.31 (0.18)	0.24 (0.14)	0.1347
Width of second band at distal attachment point	0.61 (0.50)	1.45 (0.75)	0.1116	0.81 (0.48)	1.10 (0.91)	0.8597
Thickness of second band at distal attachment point	0.08 (0.07)	0.18 (0.10)	0.2159	0.09 (0.07)	0.15 (0.11)	0,5959
Length of main band or length to split in Y type	3.10 (1.13)	3.29 (0.99)	0.4508	3.34 (1.04)	2.93 (1.06)	0.1106
Lenght of second band	3.01 (1.01)	3.99 (1.17)	0.5959	2.53 (0.71)	4.11 (0.86)	0.1116
Lenght of posterior arm to split point of the ligament in Y type	1.60 (0.41)	1.35 (0.53)	0.5708	1.71 (0.34)	0.98 (0.01)	0.0189
Lenght of anterior arm to split point of the ligament in Y type	2.47 (0.90)	2.39 (1.16)	0.9247	2.79 (0.86)	1.51 (0.32)	0.0827

*R* right side, *L* left side, *F* female, *M* male.

*Significant p according to Bonferroni correction is 0.005.

## Discussion

This study presents the first systematic classification of the CFL in human fetuses; previous studies have presented a similar classification, but for adult cadavers.

During the fourth week of embryonic development, the lower limb buds become visible for the first time, and 2 weeks later the distal portion of the limb bud develops into the digital plate. During this period, the plate is oriented in line with the long axis and the entire limb is externally rotated. The plantar surface of the digital plate is thus turned towards in a cranial direction. Then the digital plate adopts a fan-like shape appearing from the eighth-week notches. At the same time, during the eighth week of embryonic development, external rotation decreases, ending with the plantar surfaces being turned to each other and the feet in a position of equino-varus-adductus. Finally, at the end of the eleventh week of fetal life, the position of the feet is close to neutral. Joint cavities with synovial linings appear after 10 weeks of fetal life. There are two ways of developing the collateral ligaments of joints: by localized thickening of the joint capsule or as a loose tissue distant from the capsule or joint cavity. After ten weeks of gestation age, the CFL, ATFL and PTFL begin to form. ATFL arises as a thickening of the joint capsule, while the ATFL and CFL are distant from the capsule. At this stage, histological studies describe these ligaments as consisting of straight fibers. With further embryological development, the fibers in the ATFL and PTFL change direction, while those of the CFL simply elongate. Therefore, we believe that the presence of additional bands, and their shape, are determined from the beginning of fetal life. Indeed, no significant difference in CS length was found between types in the present study.

The calcaneofibular ligament originates on the anterior part of the lateral malleolus. It is placed beneath the inferior band of the ATFL. Recent anatomical studies indicate that the anterior part of CFL and inferior part of the ATFL are covered by a fibrous layer; however, this is only superficial and easy to remove. After removing the fibers, the ATFL and CFL have independent attachments^5^. In the plantigrade position, the ligament runs obliquely downwards deeply to fibular longus and fibularis brevis tendons and sheaths^6^. They also most completely cover the CFL, and about 1 cm of the ligament is uncovered^7^. The CFL inserts to the small tubercle at the posterior aspect of the lateral calcaneus^8^. An anatomically positioned CFL forms a 113° to 150° angle with the fibula^9^.

Although several studies describe morphological variations of the CFL, all are based on adult cadavers. Burks et al.^9^ described he CFL as band-shaped ligament with a mean length of 35.8 mm; although the study mentioned the LTC ligament it did not give a precise frequency of occurrence^9^. Trouilloud et al.^10^ present a classification of three types of CFL ligament based on its relationship with the TCL, without describing the shape of the main band. Wiersma et al.^11^ propose two variants of CFL shape: a cord-like structure (66%) and a flat, fanning shape (34%). Kitsoulis et al.^12^ distinguished three types of CFL based on the presence of an accessory band: one band was present in 52 out of 72 cases, two bands in sixteen cases and three bands in four lower limbs. The most recent current classification was given by Pereira et.al.^13^ from a study of 47 adult human limbs, comprising single bundle, V-shape, Y-shape and double band CFL types^13^. It is important to note that these findings closely resembleour proposed classification; however, we propose expanding the Y-shape and double band categories, with the latter including subtypes based on origin point^13^.

We proposed a first CFL classification based on human fetuses. Due to the fact that between the types there was no significant statistical difference regarding the cranio-sacral length, we believe that the type of CFL is already defined in fetal life. In our opinion, the shape of the CFL is determined from the beginning and it is observed in other human ligamentous structures, such as the ligamentum mucosum^14,15^. Performing studies on a larger sample of adult lower limbs should confirm our classification of CFL types. Particularly noteworthy are the types 2 and 4 proposed by us. In our opinion, type 4a is a previously undescribed double CFL. Cases of a double band ligament, which in classical anatomy are described as a single band, are known in current anatomical literature for example the double fibular collateral ligament of the knee^16^. There are morphological similarities between types 2b and 4b due to the connection with the talus bone. The occurrence of an independent ligament attaching to the talus bone is described in the literature in 23–35% of cases^10,13^. Pereira et al.^13^ in his work on adults described both of the above-mentioned types and suggested that the Y-type, which corresponded to our type 2b, is formed by CFL and LTC blended into a single ligament. In our work, we proved that this assumption is wrong because these ligaments already have that shape during fetal life. In our opinion, these are two different morphological types, however, the Y-shape with attachment to the talus bone certainly increases the stability of the ankle joint. Another interesting fact is that the occurrence of LTC has always been associated with the occurrence of single band CFL.

Recent anatomical literature indicates that the ATFL and CFL are components of the lateral collateral ligament complex^17^. The inferior ATFL band is connected by arciform fibers to the CFL; this forms an extra-articular part creating the LFTCL. Interestingly, these fibers are visible during fetal growth. In contrast, the superior band of ATFL follows an intraarticular course. In ankle inversion sprains, this intraarticular part is ruptured most easily, followed by the CFL and inferior band of ATFL^17–19^.

This study has some limitations. A larger sample size would have been desirable; however, it is the first classification of the ligament in human fetuses. In addition, all samples were obtained from a specific population from Lodz, Poland; as such more studies are needed to determine ligament types in larger populations. In addition, no calculation of the sample size was carried out. Nevertheless, our study is the first classification based on human fetuses. We believe that a systematic classification will be a valuable tool to improve knowledge about embryology of the ankle joint region.

## Conclusion

We distinguished four types of CFL in human fetuses. The most common was Type I, characterized by a band shape. The results of our study on the anatomical variation of CFL in fetuses will allow further comparisons with other studies focused on ankle joint development, including this performed on adult cadavers.

## Data Availability

Please contact authors for data requests (Łukasz Olewnik—email address: lukasz.olewnik@umed.lodz.pl).
